# Does a Bevacizumab-based regime have a role in the treatment of children with diffuse intrinsic pontine glioma? A systematic review

**DOI:** 10.1093/noajnl/vdac100

**Published:** 2022-06-24

**Authors:** Mia Evans, Ria Gill, Kim S Bull

**Affiliations:** Faculty of medicine, University of Southampton, Southampton, UK; Faculty of medicine, University of Southampton, Southampton, UK; Clinical and Experimental Sciences, University of Southampton, Southampton, UK

**Keywords:** Bevacizumab, diffuse intrinsic pontine glioma, quality of life, survival, systematic review

## Abstract

**Background:**

There are no effective treatments for diffuse intrinsic pontine glioma (DIPG); median survival is 11.2 months. Bevacizumab has the potential to improve quality of life (QOL) and survival in DIPG but has never been evaluated systematically. The aim of this review was to assess Bevacizumab’s role in the treatment of DIPG.

**Methods:**

MEDLINE, EMBASE, Scopus, and Web of Science were searched for relevant studies using terms developed from alternatives for Bevacizumab and DIPG. One reviewer screened titles and abstracts, then two reviewers screened full texts. Data were extracted into tables and quality assessed using methodological index for non-randomized studies and JBI tools.

**Results:**

Searching revealed 1001 papers; after deduplication 851 remained. After screening of titles and abstracts, then 28 full texts, 11 studies were included. Four studies reported a median overall survival longer than historical data, however, two found no significant impact of Bevacizumab. Five studies reported a radiological response in a proportion of participants and two reported no response. Three studies, evaluating clinical response, reported improvement in a proportion of patients. Three studies, evaluating QOL, reported stability or improvement. Four studies, evaluating steroid use, reported reductions in the proportion of patients receiving steroids. In radiation necrosis treatment, Bevacizumab led to clinical improvement in 6/12 patients in 2 studies and permitted a reduction in steroid use in most patients.

**Conclusions:**

Insufficient evidence means the role of Bevacizumab in the treatment of DIPG is unclear. However, Bevacizumab may be beneficial to some patients. The review highlights the need for further research in this area.

**Key Points**

- Due to insufficient evidence the role of Bevacizumab in DIPG treatment is unclear.
- However, Bevacizumab has high tolerability and may improve QOL and reduce steroid use.
- There is a need for further research in this area, particularly randomized controlled trials.

**Importance of the Study**

Diffuse intrinsic pontine glioma (DIPG) is a highly aggressive brainstem tumor. It is the leading cause of brain-tumor-related death in childhood and there are currently no effective treatments. After scoping the literature, it was clear that there were conflicting reports on the role Bevacizumab has in the treatment of DIPG. Further, no systematic reviews had been completed in this area. After conducting a systematic literature review, due to insufficient evidence, it was not possible to reach a definitive conclusion regarding the beneficial role Bevacizumab has in the treatment of DIPG. However, there is evidence to suggest that Bevacizumab may be beneficial to some patients. This review highlights the need for further research in this area, specifically in the form of randomized controlled trials.

Brain tumors are the leading cause of cancer-related death in children aged between 1 and 19 years.^1,2^ They are also the most common form of cancer in children aged less than 15 years.^1,2^ Diffuse intrinsic pontine glioma (DIPG) constitutes 15%–20% of all pediatric CNS tumors and is the leading cause of brain-tumor-related death in childhood.^3,4^ It is a highly aggressive pediatric brain tumor, although there have been some rare cases in adults,^5^ carries an extremely poor prognosis.^6^ There are no effective treatments and no chance of survival.^7^ The median age at diagnosis is estimated to be between 6 and 7 years^3^ and median survival is currently 11.2 months,^8^ with more than 90% of children dying within 2 years of diagnosis.^3,9^ The outlook of childhood cancer is constantly improving; the 5-year survival rate is now 84%^10^ but there have been no improvements in the prognosis of DIPG for over 30 years since the introduction of radiotherapy.^3^ More effective treatments are desperately needed.^9^

In the WHO 2016 classification, DIPG was reclassified as a subtype of diffuse midline glioma, a midline astrocytoma that occurs due to a specific mutation in the H3F3A gene (K27M), the vast majority of DIPG tumors contain this mutation.^11^ Reasons for the poor prognosis include that: it develops in the pons which is essential for blood pressure, heart rate, breathing, and bladder control; nerves controlling vision, movement, speech, hearing, and swallowing also all pass through or near the pons^12^; DIPG is also highly aggressive and fast-growing; and the tumor is inaccessible surgically due to its position and diffuse nature.^3,12^ Treatment by total surgical resection is not an option and Gallitto et al.^13^ concluded that overall survival (OS) was not improved and may be worsened by carrying out subtotal resection.

The standard treatment for DIPG is conventionally fractionated daily photon beam radiotherapy for 6 weeks which slows tumor growth temporarily in 80%–90% of patients.^13,14^ Other radiotherapy regimens, such as altered fractionation, have not led to significant improvement in outcomes over conventional radiotherapy^14^ and overall, neither has chemotherapy.^15^ Due to the high selectivity of the blood-brain barrier, it is thought that only a small number of chemotherapy drugs are able to reach the tumor.^15,16^ Additionally, DIPG tumor cells appear to be resistant to chemotherapy.^16^ However, some early trials of chemotherapy regimens with Temozolomide and/or Irinotecan both showed improved survival outcomes.^17–19^ In DIPG, Dexamethasone is often given to reduce neurological symptoms caused by the tumor^12^ and for the symptomatic treatment of radiation necrosis.^20^ It reduces cerebral edema which causes headaches, nausea, weakness, and problems walking, due to increased intracranial pressure.^12,21^ However, steroids have many side effects including increased appetite, edema, changes in behavior, muscle weakness, immunosuppression, acne, and difficultly sleeping.^12,20^ These symptoms adversely affect the quality of life (QOL) of children with DIPG. It is, therefore, important to consider whether the side effects of steroids outweigh the benefits, especially when higher doses are required at tumor progression.^12,20,22^

Bevacizumab is a targeted antiangiogenic agent.^23^ It is an anti-vascular endothelial growth factor (VEGF) monoclonal immunoglobulin G (IgG) antibody that works by binding to and inactivating VEGF, a protein expressed by tumor cells that stimulates tumor angiogenesis and growth.^23^ Therefore, Bevacizumab inhibits the formation of new blood vessels within the tumor, thereby reducing blood supply and preventing growth.^21,23^ Bevacizumab is likely to be of particular benefit in DIPG as messenger RNA profiling studies have exposed an overexpression of VEGF in DIPG compared to normal brain tissue and in adults and children with high-grade glioma.^6,24^ Bevacizumab is not currently approved for use in DIPG in the UK, but in 2009 it received accelerated approval by the US Food and Drug Administration for the second-line treatment of glioblastoma multiforme in adults after it demonstrated durable objective responses.^25–27^ Bevacizumab is generally licensed for use within chemotherapy regimens rather than alone,^23^ for example, alongside Irinotecan and Temozolomide.^17–19,28^ It is given intravenously, and potential toxicities include fatigue, hypertension, thrombocytopenia, neutropenia, proteinuria, cerebral ischemia, and impaired wound healing.^18,29^ However, severe toxicities are uncommon, and the good tolerability associated with Bevacizumab-based regimens has been well documented.^17–20,28–31^ Bevacizumab has many proposed roles in the treatment of DIPG such as improving survival and QOL and reducing cerebral necrosis.^17–20,23,28^

The HERBY trial (phase II)^32,33^ evaluated the role of Bevacizumab in addition to temozolomide/radiotherapy in children with non-brainstem high-grade glioma including non-brainstem K27M tumors. Grill et al.^32^ determined that Bevacizumab did not improve event-free survival (EFS) in this cohort, which presents the question if the same is true for brainstem K27M tumors such as DIPG.

There is limited data and agreement on the beneficial role Bevacizumab has in the treatment of DIPG, and currently, as far as we are aware, no systematic review has been published on the subject. Therefore, we aimed to collate the evidence and assess the role of this drug in the treatment of DIPG.

## Methods

A scoping search was conducted in January 2021 to assess the availability of papers on this topic, to gain some background information, and to establish an appropriate review question.

The systematic review was undertaken in accordance with PRISMA guidelines^34^ but was not registered on PROSPERO. A review protocol was developed between September 07, 2021 and September 10, 2021 (available upon request).

### Search Strategy

Four electronic databases EMBASE (*Embase Classic + Embase 1947 to 2021 September 17)*, Web of Science, Scopus, and MEDLINE (*Ovid MEDLINE(R) 1946 to September Week 2 2021)* were searched on September 20, 2021.

Search terms were developed by compartmentalizing the topic into key concepts using the patient, intervention, comparison, outcome (PICO) framework.^35^ Alternative words for both DIPG and Bevacizumab were included. Both free-text terms and subject headings (medical subject headings [MeSH] terms) were used. Children were not specified in the population at this stage, as some key papers included young adults over 18 years as well as children. Papers that included only adults were removed during screening. Truncation was used to ensure plurals were included as well as both English and American spellings. To ensure all relevant studies were included, no search terms relating to a comparator were used, as studies with or without a comparator were both included. Due to the small number of papers encompassing both DIPG and Bevacizumab, any outcome was included at this stage, then during screening and selection only studies reporting outcomes stated in the eligibility criteria were included. The results of an example search are presented in Supplementary Table S1. Forward and backward chain searching using Web of Science was conducted on full-text papers that were included in the review.

### Eligibility Criteria

#### Inclusion criteria

1. Diagnosis of DIPG; other brain tumors included if the analysis was distinct and separate
2. Any treatment regime which included Bevacizumab as part of the primary intervention for DIPG at diagnosis, at progression, and as treatment for radionecrosis
3. Outcomes included: OS, progression-free survival (PFS)/EFS, time to progression, QOL, clinical/neurological response, radiological response, or change in steroid use
4. Primary research papers of all study types
5. English language and any year

#### Exclusion criteria

1. Brain tumor studies in which DIPG was not included or where DIPG was included but not analyzed separately
2. Adult-only studies (>18 years)
3. Bevacizumab was not a primary treatment
4. Grey literature
5. Reviews and meta-analyses
6. Qualitative studies

### Study Screening and Selection

Search results were imported into EndNote 20^36^ and deduplication was conducted. The resulting papers were imported into Rayyan QCRI^37^ for screening and selection. First, titles and abstracts were screened against the eligibility criteria independently by Reviewer 1 (M.E.) and were grouped into included, excluded, or maybe categories. If in any doubt, the reviewer included the paper in the maybe group to be sure that none were missed. Full-text articles were obtained for the next stage which involved screening the resulting full-text papers using a screening and selection tool. This was conducted independently by Reviewers 1 (M.E.) and 2 (R.G.). Any disagreements were discussed and resolved with potential input by the supervisor (K.S.B.).

### Data Extraction

Four detailed data extraction tables (available on request) were created: (1) study characteristics; (2) patient characteristics; (3) results of treatment for DIPG; and (4) results of treatment for radiation necrosis in patients with DIPG. Papers were printed, extractable data were highlighted, and the detailed data extraction tables were then completed. Tables were piloted on the first three studies by Reviewer 1, and additions or exclusions made, where necessary, to ensure all relevant data were collected. Once data extraction was completed by Reviewer 1, tables were cross-checked by Reviewer 2 and a week later by Reviewer 1 again to ensure inter- and intra-rater reliability. Disagreements were discussed and, if appropriate, tables amended. These detailed data extraction tables were used to complete summary data tables for use in the review. Data extraction was completed before the quality assessment to minimize reporting bias.

### Quality Assessment

Quality assessment was completed by Reviewer 1. The methodological index for non-randomized studies (MINORS)^38^ tool was used to assess the quality of all studies apart from case reports. After piloting using the first three papers, the criteria were modified in two ways: (1) the addition of a question on whether the intervention was standardized, as non-standardized interventions were deemed to be of lower quality, and (2) to improve the appropriateness of the criteria for retrospective studies, the wording of criteria 2, 3, and 8, was modified. Each item was scored 0 (not reported), 1 (reported but inadequate), or 2 (reported and adequate) (Supplementary Figure S1). Quality assessment of case reports was completed using the JBI critical appraisal checklist for case reports.^39^ Questions were answered yes, no, or not clear (Supplementary Figure S2.). Both tools were recommended by Ma et al.,^40^ the MINORS for non-comparative non-randomized studies and the JBI for case reports.

## Results

### Search Results

**Figure 1.**
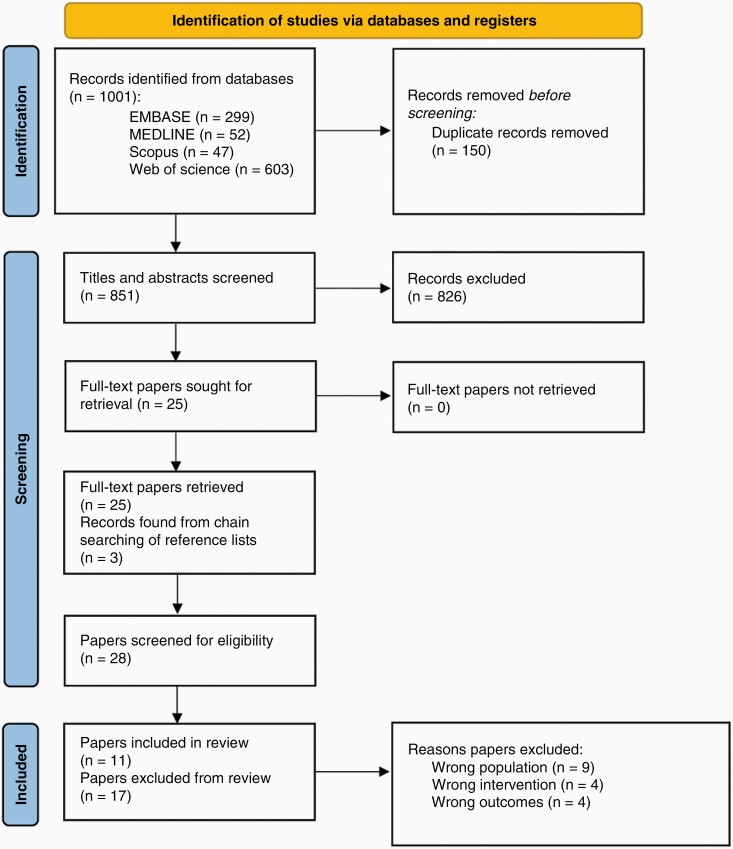
PRISMA flow diagram presenting search process.

Searching of the electronic databases resulted in 1001 potential papers and of these, 851 remained after deduplication. Title and abstract screening of these resulted in the retrieval of 25 full-text papers on which forward and backward chain searching was conducted on the references lists. This resulted in three further papers being obtained. Therefore, 28 full-text papers underwent screening and selection by Reviewers 1 and 2 independently, resulting in 82.1% agreement. After discussion, 11 papers were included in the review (Figure 1). Reasons for exclusion of the remaining 17 papers are presented in Supplementary Table S2.

### Study and Patient Characteristics

Of the 11 included studies (Table 1), 4 were multi-institutional and the remaining 7 were single-institutional. Eight studies took place in the United States, 1 in the Netherlands,^17^ 1 in Japan,^41^ and the remaining study^42^ was multi-institutional across Canada, Argentina, Czech Republic, Spain, and Australia. Across the studies 3 were retrospective,^18,19,42^ 2 were case reports,^20,43^ and the remaining 6 were phase I or II clinical trials. All data were collected between 1995 and 2018, with a total of 97 patients across all studies. Of the 11 studies, 9 evaluated the role of Bevacizumab as a treatment for DIPG, and the remaining 2 studies (study 2^42^ and 7^20^) as a treatment for radionecrosis in children with DIPG. Interventions varied; treatments alongside Bevacizumab included radiotherapy, chemotherapy (temozolomide, irinotecan, carboplatin, and etoposide), erlotinib, valproic acid, and Cetuximab, but all included Bevacizumab as a primary treatment as per the eligibility criteria. Bevacizumab was administered intravenously in 10 studies, and in 1, intraarterially.^6^ A Bevacizumab dosing regimen of 10 mg/kg every 2 weeks was given in 9 of 11 studies, another study gave 15 mg/kg every 3 weeks, and a final study gave a one-off dose of 15 mg/kg intraarterially.

**Table 1.** Study Characteristics

Study No.	Author (Year)	Title	Country	Years Data Obtained	*N*	*n*_(DIPG)_	Study Design	Intervention
1	Aguilera (2013)^43^	Prolonged survival after treatment of diffuse intrinsic pontine glioma with radiation, temozolomide, and bevacizumab: Report of 2 cases	USA	2008–2009	3	2 (1 patient not included in case reports, unclear why)	Case report	RT then BEV (10 mg/kg every 2 weeks) and TMZ, five weeks after completion
2	Baroni (2020)^42^	Bevacizumab for pediatric radiation necrosis	Canada, Argentina, Czech Republic, Spain, and Australia	NR	26	8	Retrospective	BEV (10 mg/kg for 7 patients and 5 mg/kg for 1 every 2 weeks) for the treatment of radiation necrosis
3	Crotty (2020)^18^	Children with DIPG and high-grade glioma treated with temozolomide, irinotecan, and bevacizumab: the Seattle Children’s Hospital experience	USA	2009–2018	26	10	Retrospective	RT with TMZ, followed by a maintenance regime of TMZ, IRO, and BEV (IV 10 mg/kg every 2 weeks)
4	El-Khouly (2021)^17^	A phase I/II study of bevacizumab, irinotecan and erlotinib in children with progressive diffuse intrinsic pontine glioma	Netherlands	2011–2018	9	9	Phase I/II	BEV (10 mg/kg biweekly), IRO and Erlotinib
5	Gururangan (2010)^29^	Lack of efficacy of bevacizumab plus irinotecan in children with recurrent malignant glioma and diffuse brainstem glioma	USA	2006–2008	31	16	Phase II	BEV (10 mg/kg every 2 weeks) and IRO
6	Hummel (2016)^28^	A pilot study of bevacizumab-based therapy in patients with newly diagnosed high-grade gliomas and diffuse intrinsic pontine gliomas	USA	2009–2013	27	15	Pilot	RT with BEV (10 mg/kg on days 1, 15, 29, 43) then BEV (10 mg/kg every 2 weeks) and IRO as maintenance therapy 4 weeks after RT completion
7	Liu (2009)^20^	Bevacizumab as therapy for radiation necrosis in four children with pontine gliomas	USA	1995–2008	4	3	Case studies	BEV (10 mg/kg every 2 weeks)
8	McCrea (2021)^6^	Intraarterial delivery of bevacizumab and cetuximab utilizing blood-brain barrier disruption in children with high-grade glioma and diffuse intrinsic pontine glioma	USA	2013–2018	13	10	Phase I	A one-time intraarterial dose of BEV (15 mg/kg) and Cetuximab after BBB disruption with Mannitol
9	Okada (2013)^41^	Phase I study of bevacizumab plus irinotecan in pediatric patients with recurrent/refractory solid tumors	Japan	2009–2011	11	2	Phase I	BEV (10 mg/kg) plus IRO every 2 weeks
10	Su (2020)^44^	A phase 2 study of valproic acid and radiation, followed by maintenance valproic acid and bevacizumab in children with newly diagnosed diffuse intrinsic pontine glioma or high-grade glioma	USA	2009–2015	38	19 (16 received BEV)	Phase II	RT and valproic acid followed by maintenance valproic acid and BEV (10 mg/kg every 2 weeks)
11	Zaky (2013)^19^	Treatment of children with diffuse intrinsic pontine gliomas with chemoradiotherapy followed by a combination of temozolomide, irinotecan, and bevacizumab	USA	2007	6	6	Retrospective	Chemotherapy (carboplatin and etoposide in 5 and TMZ in 1) and RT followed by IRO, TMZ, and BEV (15 mg/kg every 3 weeks)

Abbreviations: BBB, blood-brain barrier; BEV, Bevacizumab; EFS, event-free survival; HGG, high grade glioma; IRO, Irinotecan; MR, magnetic resonance; NR, not reported; OS, overall survival; PFS, progression-free survival; QOL, quality of life; RT, radiotherapy; TMZ, Temozolomide; VEGFR-2, vascular endothelial growth factor receptor-2.

Treatment was initiated at different points in the clinical course (Table 2). In five studies treatment began at diagnosis, in three at progression/recurrence, in two at the onset of radiation necrosis symptoms, and in one it was not clear (this study was put in the treatment given in progression group as patients had received previous treatment). The role of Bevacizumab was evaluated using various outcomes including survival, QOL, clinical response, radiological response, and steroid use.

**Table 2.** Patient Characteristics

Study No	No of Included Participants	Median Age at Diagnosis (Years)	Sex (% Male)	Outcomes Measured Relevant to Review	Median Number of BEV Courses	When in the Disease Course Treatment Given	Prior Treatment
1	2	9^a^ (7–11)	50^a^	Radiological response (T2-weighted MRI) Reduction in dexamethasone dose	NR	Newly diagnosed, after RT	RT and Dexamethasone
2	8	8.6^a^	NR	Clinical response Radiological response (T2/FLAIR MRI) Reduction in dexamethasone dose^b^	4.5^a^ (4–6)	Onset of radiation necrosis symptoms	RT and Dexamethasone Likely other treatment given but not clear
3	10	10.9^b^	50^b^	Median OS Median EFS Steroid use	Median duration = 271 days	At diagnosis	No previous treatment
4	9	7.7	55.6^a^	Median OS Median secondary PFS (from initiation of study to secondary progression) QOL (using PedsQL questionnaires) Clinical/neurological response Radiological response (tumor growth on MRI)	NR	At clinical or radiological progression	At least RT 4 patients received gemcitabine (as part of first phase of trial) 1 patient re-irradiated
5	16	8.7 (2.9–14.6)	NR	Median and 6-month PFS Radiological response (median diffusion ratio, rate of sustained objective response and SD on MRI)	2 (1–12)	At recurrence or progression	RT with or without chemotherapy
6	15	6^a^ (3–26)	53.3^a^	Median OS Median EFS Health-related QOL (PedsQL for patients 5–18 years and FACT-BR for patients > 18 years) Radiological response (MRI perfusion/diffusion) Functional abilities (BOT-2 and FRESNO)	27 patients received 170 courses of maintenance therapy^b^	At diagnosis	NR
7	4	NR	75^a^	Clinical response Reduction in dexamethasone dose Radiological response (T1-weighted and FLAIR MRI)	4.5 (3–5)	Onset of radiation necrosis symptoms	RT and 2 received an investigational agent on a phase I trial
8	10	5.5^a^ (4–14)	50^a^	Clinical response (symptom improvement) Mean OS Radiological response (T1-weighted postcontrast and T2-weighted FLAIR sequences)	1 (apart from one who had two)	NR	Standard treatment, at least RT. Six patients enrolled in other clinical trials
9	2	5^a^	50^a^	Clinical response Radiological response (tumor size on MRI)	7^a^ (5–9) doses	At progression (4 and 8 months)	RT and chemotherapy
10	16	7.3^a^ (3.2–17.9)	50^a^	Median OS Median EFS and one-year EFS Radiological response (tumour size on MRI)	NR	At diagnosis	No previous treatment
11	6	6.3^a^ (3.5–10.6)	33^a^	Median OS Median EFS Radiological response (T2-weighted and/or FLAIR sequences)	7 cycles (4–11)	At diagnosis	No previous treatment

Abbreviations: BOT-2, Bruininks-Oseretsky Test of Motor Proficiency 2nd edit; DIPG, diffuse intrinsic pontine glioma; EFS, event-free survival; FACT-Br, Functional Assessment of Cancer Therapy—Brain; MRI, magnetic resonance imaging; NR, not reported; OS, overall survival; PedsQL, Pediatric Quality of Life; PFS, progression-free survival; QOL, quality of life; RT, radiotherapy.

^a^Analysis not separate from other patients in trial that did not have a diagnosis of DIPG.

^b^Statistical analysis carried out by reviewer.

### Role of Bevacizumab in Improving Survival in Patients With DIPG

Six studies evaluated if Bevacizumab had a role in improving survival, with differing conclusions (Table 3). Studies 3, 4, 8, and 11 reported that Bevacizumab led to a median OS longer than historical data. With studies 3 and 11, where Bevacizumab was given at diagnosis, also reporting an increased EFS. However, studies 6 and 10 where Bevacizumab was also given at diagnosis, reported no significant impact on median OS or EFS. Study 5 reported a median PFS of 2.3 months, concluding that Bevacizumab had minimal efficacy.

**Table 3.** Results of Treatment for DIPG

Study No	Median OS (Months)	Median EFS/PFS (months)	Radiological Response	Clinical/Neurological Response (Symptom Improvement)	QOL	Steroid Use (Reduction in Duration/Dose)	Feasibility, Safety, and Tolerability	Conclusion
Results of treatment for DIPG at diagnosis								
1	Patient not in case reports had an OS of 14 months^a^	At point of writing PFS 37 and 47 months.^a^ Patient not in case reports had PFS of 12 months^a^	65% and 80% reduction in tumor size on T2-weighted imaging	NR	Excellent	Discontinued 6 and 10 weeks after completion of radiation	Feasible and well tolerated	PFS rate encouraging significant reduction in tumor size and no steroids required after 10 weeks
3	13.3^a^	EFS = 9.3^a^	NR	NR	NR	22% were receiving steroids at the initiation of maintenance therapy and 3% at start of maintenance course 6^b^	Demonstrates tolerability	Superior survival to nearly all other published treatment strategies Improved 1-year OS of 80% (historically 45.3% using International DIPG Registry)
6	10.4^c^ (6.8–16.9)	PFS = 8.2^c^	Baseline tumoral enhancement noted in 12/15 patients	NR—no patients completed functional outcome assessment	General fatigue and brain-tumor-specific QOL measures remained stable or improved over time	NR	Feasible, safe and tolerable	No significant impact on median OS
10	10.3^a^ (11.4^e^ when 16 patients)	EFS = 7.8^d^ (8.0^e^ when 16 patients) One-year EFS = 12%	7/16 had sustained PRs beyond week 22. Every patient had either a PR or MR (<50% reduction in tumor size).	NR	NR	NR	Well tolerated	Did not appear to improve EFS or OS OS and EFS were comparable to other recent collaborative trials but not statistically superior Encouraging tumor responses
11	14.67^a^ 1-year OS = 67%	EFS = 10.43^a^	4/6 patients had PRs, rest had SD, in response to chemoradiotherapy. Overall responses sustained by maintenance therapy until progression	NR	NR	NR	Increased but acceptable toxicity	Modest increase in EFS and OS compared to published literature
Results of treatment for DIPG at progression/recurrence								
4	13.8^a^ (9.3–33.0)	Secondary PFS = 3.2^d^ (1.0–10.9)	At three months, PR observed in three patients, SD in one, and PD in five. At 6 months PD observed in two, and SD in two	Stable during the first 3 months of treatment in 4/9 patients (5 showed progression)	Stable; was not significantly different between time points. Slight reduction when considering physical performance, nausea and fear of procedures/treatments	NR	Safe and well-tolerated	Median OS longer than known form literature, including survival data of patients receiving radiotherapy only but not powered on efficacy QOL stable
5	NR	Median PFS = 2.3^d^ 6-month PFS = 9.7%^d^	Median diffusion ratio increased by 6.9% (−39% to 53%) No sustained objective responses were observed. Sustained SD (>12 weeks) was observed in 5/16 patients for a median of 126 days	NR	NR	NR	Minimal efficacy but well-tolerated	PhosphoVEGFR-2 levels reduced (pharmacokinetic response) Not effective in producing sustained objective responses, with most patients coming off study for PD Rate of disease stabilization appears indifferent from standard chemotherapy May work better at time of initial diagnosis
8	17.3^a^ (519 days [221–761])	NR	Areas of contrast uptake seen in all patients. T1-weighted postcontrast volume reduced in 9/10 patients post-procedure day 1 suggesting treatment reached tumor. On the 1-month post-procedure T1-weighted postcontrast 3 patients had PD, 2 SD, 2 had PR and 1 complete response. Post-procedure day 1, 8 patients exhibited SD and 2 progressive disease on T2-weighted FLAIR. At 1 month 3 had SD and 5 PD.	5/8 with symptoms had subjective symptom improvement. 2 patients were able to decrease steroid dose and go back to school Symptom improvement lasted approximately 1 month in all^b^	NR	Some patients were able to reduce steroid dose or wean off steroids completely, but exact numbers were not given	Well-tolerated Results demonstrate safety	OS longer than historical controls
	NR	NR	Patient 1 = SD at end of treatment (9 doses) Patient 11 = PD 5 doses in no radiographic response in either	Both patients experienced improvement of clinical/neurological symptoms	NR	Patient 1 was able to taper steroids	Well-tolerated and has some antitumor activity Acceptable safety during short-term treatment	Both had neurological improvement, may result from reduction of tumoral edema Objective response observed

Abbreviations: EFS, event-free survival; MR, minor response; NR, not reported; OS, overall survival; PD, progressive disease; PFS, progression-free survival; phosphoVEGFR-2, vascular endothelial growth factor receptor-2 phosphorylation; PR, partial response (>50% reduction in tumor size); QOL, quality of life; SD, stable disease.

^a^From diagnosis.

^b^Analysis not separate from other patients in trial that did not have a diagnosis of DIPG.

^c^From start of any treatment.

^d^From initiation of study.

^e^Analysis when only children treated with bevacizumab included.

### Role of Bevacizumab in Producing a Radiological Response

Studies 1, 4, 5, 6, 8, 9, 10, and 11 all evaluated the role of Bevacizumab in producing a radiological response (Table 3). This was calculated in a variety of ways including median diffusion ratio, T1-weighted imaging, T2-weighted imaging, tumoral enhancement, and tumor measurements on MRI. Criteria were used to categorize the response in most papers into partial response (PR), minor response (MR), stable disease (SD), and progressive disease (PD). In studies 1, 6, 10, and 11 Bevacizumab was given at diagnosis; in study 1, the 2 patients had a 65% and 80% reduction in tumor size on T2-weighted imaging. Study 10 reported that 7 of 16 patients had sustained PRs (>22 weeks) and every patient experienced an MR (<50% reduction in tumor size) or PR after treatment at diagnosis. In study 11, maintenance therapy sustained the radiological responses due to chemoradiotherapy until progression. Studies 4, 5, 8, and 9 evaluated survival when Bevacizumab was given at progression. Study 4 investigated the role of Bevacizumab, erlotinib, and irinotecan at tumor progression. After 3 months of treatment, 3 patients had PRs (>50% reduction in tumor size), 1 had SD, 5 had PD. By 6 months, 2 patients remained with SD. Study 5 investigated Bevacizumab and irinotecan at tumor progression and concluded that no sustained objective responses were observed radiologically, and sustained SD (>12 weeks) was observed in 5 of 16 patients. Study 8 measured radiological response using T1- and T2-weighted contrast imaging. One month post-procedure T1-weighted showed 1 complete response, 2 PR, 2 SD, and 3 PD, whereas T2-weighted imaging showed three SD and 5 PD. Study 9 reported no radiographic response in either patient, one had SD at the end of treatment and the other had PD, 5 of 9 doses into treatment.

### Role of Bevacizumab in Symptom Improvement (Clinical/Neurological Response), QOL, and Reduction in Steroid Use

Studies 4, 8, and 9 all evaluated whether a Bevacizumab-based regime led to improvements in clinical/neurological response at progression/recurrence (Table 3). In study 4, four of nine patients remained stable during the first 3 months of treatment after receiving Bevacizumab for tumor progression. In study 8, 5 of 8 patients with symptoms had subjective symptom improvement after treatment; this included 2 patients who were able to return to school. Study 9 included 2 patients with tumor progression; both experienced improvement of clinical/neurological symptoms after treatment with Bevacizumab and Irinotecan.

Studies 1 and 6 investigated whether Bevacizumab had a role in improving QOL at diagnosis, with both studies reporting QOL improved during treatment. In study 4, Bevacizumab was given at progression, overall QOL was reported to remain stable throughout treatment.

Bevacizumab may have a role in the reduction of steroid use. Study 1 included 2 case reports of long-term survivors treated with Bevacizumab and Temozolomide. Steroids were discontinued 6 and 10 weeks after completion of radiotherapy. Study 3 reported that 22% of patients received steroids at the beginning of maintenance therapy, and this was reduced to 3% after 5 courses. At least 2 patients were able to decrease steroid dose in study 8, but steroid use was not evaluated as an outcome. Of the 2 patients with DIPG in study 9, 1 was able to reduce steroid use after Bevacizumab treatment.

### Role of Bevacizumab in Treatment of Radiation Necrosis in Children With DIPG

Study 2 retrospectively analyzed the medical records of children treated with Bevacizumab for radiation necrosis across 5 institutions (Table 4). Eight patients with DIPG were included, of these, three experienced clinical improvements, four remained stable, and one progressed. Of the 5 patients where radiological response was assessed, 2 had a reduction in MRI response, and 3 remained stable. The regime permitted a reduction in steroid dose and/or duration in most patients. It was concluded that Bevacizumab was safe, very well-tolerated, and effective in a proportion of patients.

**Table 4.** Results of Treatment of Radiation Necrosis in Patients With DIPG

Study No.	Radiological Response	Symptom Improvement	Reduction in Dexamethasone Dose	Feasibility, Safety, and Tolerability	Conclusion
2	2/5 had reduction in MRI response and the rest stable	3/8 had clinical improvement, 1 had progression and the rest stable	18/26 patients were able to taper their dexamethasone dose^a^	Safe and very well-tolerated	Permitted a reduction in steroid dose and/or duration in most patients Effective in a proportion of patients
7	Patient 1 = decreased enhancement in the region of necrosis Patient 2 = decrease in enhancing necrotic region and edema seen on the FLAIR sequence Patient 3 = good improvement	Patient 1 = Significant improvement in weakness Patient 2 = rapid improvement in gait and weakness Patient 3 = good improvement	All three able to discontinue steroid use	Bevacizumab was well-tolerated Provides symptom relief with minimal toxicity	Three had significant clinical improvement and were able to discontinue steroid use

^a^ Analysis not separate from other patients in trial that did not have a diagnosis of DIPG.

Abbreviations: MRI, magnetic resonance imaging; OS, overall survival.

Study 7 reviewed the records of 4 patients with DIPG treated for radiation necrosis with Bevacizumab. One patient experienced no radiological or clinical improvement; it was concluded that this was due to the patient being in progression rather than experiencing radiation necrosis. The other 3 patients experienced symptom and radiological improvement. All 3 were able to discontinue steroid use. It was concluded that Bevacizumab provided symptom relief with minimal toxicity.

### Tolerability, Safety, and Feasibility

Ten papers described the treatment as “tolerable,” “well-tolerated,” or “demonstrates tolerability” with the eleventh paper reporting “increased but acceptable toxicity” (Table 3). Five studies described the interventions as safe and a further two demonstrated feasibility. Study 7^20^ reported that Bevacizumab “provides symptom relief with minimal toxicity.”

### Quality Assessment Results

The modified MINORS criteria showed that all retrospective and follow-up studies were of good quality, with the lowest scores of 11/18, being retrospective studies (Table 5). The JBI checklist for case reports showed that study 1 rated highly, with “Yes” answered to all questions whereas 2 of 8 of the checklist items were not present in study 7 (Table 6).

**Table 5.** Results From the Application of Modified MINORS Criteria to 9 Included Retrospective and Follow-up Studies

Criteria	2. Baroni (2020)	3. Crotty (2020)	4. El-Khouly (2021)	5. Gururangan (2010)	6. Hummel (2016)	8. McCrea (2021)	9. Okada (2013)	10. Su (2020)	11. Zaky (2013)
1. Clearly stated aim(s)	2	1	2	2	2	2	2	2	2
2. Inclusion of consecutive patients including use of eligibility criteria	0	1	2	2	2	1	2	2	2
3. Prospective collection of data	1	1	2	2	2	2	2	2	0
4. Intervention standardized	1	2	2	2	2	2	2	2	1
5. Outcomes appropriate to the aim of the study	1	2	2	1	2	2	1	2	2
6. Unbiased assessment of study outcomes	2	2	1	0	0	1	0	0	2
7. Follow-up period appropriate to the aim of the study	2	2	2	2	2	2	0	2	2
8. No loss to follow up	2	2	2	2	2	2	2	2	2
9. Prospective calculation of the study size	0	0	0	1	1	0	0	2	0
Total	11	13	15	14	15	14	11	16	13

The items are scored 0 (not reported), 1 (reported but inadequate) or 2 (reported and adequate). The maximum total score is 18.

**Table 6.** Results of JBI Critical Appraisal Checklist Completion for 2 Included Case Studies

Question	1. Aguilera (2013)	7. Liu (2009)
1. Were patient’s demographic characteristics clearly described?	Yes	No
2. Was the patient’s history clearly described and presented as a timeline?	Yes	Yes
3. Was the clinical condition of the patient on presentation clearly described?	Yes	Yes
4. Were diagnostic tests or assessment methods and the results clearly described?	Yes	Yes
5. Was the intervention(s) or treatment procedure(s) clearly described?	Yes	Yes
6. Was the post-intervention clinical condition clearly described?	Yes	Yes
7. Were adverse events (harms) or unanticipated events identified and described?	Yes	No
8. Does the case report provide takeaway lessons?	Yes	Yes

## Discussion

This systematic review aimed to collate all the available evidence to evaluate the role of Bevacizumab in the treatment of DIPG. Of the 9 studies evaluating Bevacizumab’s role in the treatment of DIPG, 6 assessed Bevacizumab’s role in survival, with conflicting conclusions. Four reported an improved median OS compared to historical data and two reported no significant difference in survival. This appears to be an improved response compared to the HERBY trial in non-brainstem K27M tumors where it was concluded that Bevacizumab did not improve EFS.^32^ Radiological response was evaluated in 7 studies, with 5 reporting a response in a proportion of patients, and the remaining 2 concluding no radiological response. Three studies reported symptom improvement in a proportion of patients but there were variations in effectiveness. Three studies reported that QOL remained stable or improved during treatment. All 4 studies investigating steroid use reported a reduction. Regarding radiation necrosis, Bevacizumab led to a clinical improvement in 6 of 12 patients across 2 studies and most patients were able to reduce steroid use. Bevacizumab-based interventions were safe and well-tolerated, with either minimal or acceptable toxicities.

Differences between studies, for example, regarding treatment interventions, made comparison difficult. Across all 11 studies, only 2^20,42^ interventions were identical, both included Bevacizumab alone. Other treatments, additional to Bevacizumab, included Temozolomide, Irinotecan, Erlotinib, or Valproic acid. Therefore, there was no evidence that any benefit was due solely to Bevacizumab. The heterogeneity in outcome measures, especially in relation to radiological response, also reduced the comparability of studies.

Treatment with Bevacizumab was initiated at different points in the clinical course of DIPG, further reducing study comparability. In 5 studies treatment began at diagnosis, in 3 at progression/recurrence, in 2 at the onset of radiation necrosis symptoms, and in one it was not clear. In study 5^29^ in which Bevacizumab was given at progression, reported efficacy may have been improved if Bevacizumab had been given at diagnosis when there is minimal tumor burden. Salloum et al.^27^ recommended administering Bevacizumab as part of initial treatment, as it may have a more pronounced effect at this point due to the crucial role angiogenesis has in gliomagenesis. However, we found insufficient evidence to suggest any differences in survival when Bevacizumab was given at diagnosis, with two studies in both the at diagnosis and at progression groups reporting improved survival, and the remainder of the studies evaluating survival reporting no improvements. Due to differences in reporting of radiological response across all studies, it was difficult to compare studies and determine if Bevacizumab led to improved radiological responses at diagnosis compared to at progression. However, in the 2 studies which reported no radiological response in all patients Bevacizumab was given at progression. Regarding QOL, giving Bevacizumab at diagnosis may also lead to greater improvements, as reported by studies 1 and 6, whereas in study 4 where Bevacizumab was only given at progression, QOL remained stable but did not improve, although improvement is unlikely to be expected in a child in decline with disease burden. However, firm conclusions regarding the benefit of Bevacizumab cannot be made from this limited number of studies that evaluated QOL.

Inconsistencies in study findings further reduced our ability to determine overall conclusions on each of Bevacizumab’s potential roles. For example, study 10^44^ reported partial radiological responses (PRs) and MRs in every patient, and in study 11,^19^ 4 of 6 had PRs, whereas, study 9^41^ reported no radiological response in either patient. Reasons for variations in effectiveness could include differences in treatment; whether treatment was given at diagnosis or progression; whether Bevacizumab was given intravenously or intraarterially; and the genetic landscape of the tumors themselves. Each tumor expresses different drug targets so respond differently, thus it can be hypothesized that tumors with overexpression of VEGF are more likely to respond to Bevacizumab treatment.^6^

Currently, Bevacizumab is not licensed for use or used commonly in the treatment of children with DIPG, this is likely due to the limited evidence assessing its role in this patient group. However, the observed improvements in symptoms, QOL, and steroid use, along with the high tolerability and good safety, suggest that Bevacizumab may have a role in clinical support. Promising results regarding its efficacy in the treatment of radiation necrosis in patients with DIPG suggest Bevacizumab may also have a role in this area. However, with only two small studies evaluating this, definitive conclusions cannot be made. The beneficial role of Bevacizumab in the treatment of radionecrosis across all brain tumor types has been documented, within a systematic review by Delishaj et al.^45^ concluding improvement in clinical and radiographic response and reductions in steroidal therapy. Khan et al.^46^ also reported radiographic response and clinical improvement without serious adverse events when Bevacizumab was used for the treatment of radiation necrosis in patients with brain metastatic disease.

Other potential roles of Bevacizumab include as adjuvant therapy with reirradiation. There is evidence to suggest reirradiation has a role in DIPG treatment with improvements in survival, symptoms, and radiological response reported across multiple studies.^47–49^ Using Bevacizumab as adjuvant therapy with reirradiation has been documented in other tumor types for example in high-grade glioma. Flieger et al.^50^ observed an increased post-recurrence survival in patients treated with re-irradiation and Bevacizumab compared to re-irradiation alone. However, there have been no trials evaluating this treatment regime in DIPG. This presents a further area for potential research regarding Bevacizumab’s role in DIPG.

For Bevacizumab to receive approval, randomized controlled trials (RCTs) and phase III clinical trials need to be conducted in this population to gather more information on its safety and efficacy to a higher degree of reliability.^51^ RCTs produce the highest quality evidence before systematic reviews and when properly conducted provide unbiased conclusions.^52^ No RCTs have been conducted in this area. Potential reasons include the low incidence rate of DIPG, low life expectancy, poor funding, and the ethical problems associated with including a control group. All the studies included in this review were either case studies, retrospective studies, or non-randomized phase I/II clinical trials. Case reports provide the lowest quality evidence according to the hierarchy of evidence pyramid, due to their focus on individual patients.^53,54^ Therefore data collected from case reports are not generalizable so cannot be used to establish cause and effect.^53^ Retrospective reports are also of lower quality evidence and scored lower on the MINORS quality assessment tool. Weaknesses of retrospective studies include no control over data collection, interventions not standardized, and high risk of bias.^51^

All included studies had limited patient numbers, with *n*_(DIPG)_ ranging from 2 to 19. Only one paper included a power calculation resulting in a sample of 19, which was met.^44^ This was the largest sample size of the included papers; therefore, it is unlikely any other study had a sample size large enough to provide sufficient power. Consequently, meaningful effects cannot be detected, and may lead to bias, therefore results may be unreliable. For a large sample size to be met, multiple institutions across Europe or globally will inevitably need to be involved.

Similar patient demographics were reported across the 11 studies. Median age at diagnosis was comparable and ranged from 5.5 to 9 years, which reflects the peak in DIPG diagnoses in mid-childhood, with the median age at diagnosis between 6 and 7 years.^3^ Gender ratios were relatively well balanced, with seven studies reporting around 50% males which is representative of the equal proportion of males and females diagnosed with DIPG.^55^ A further two studies with 75% and 33% males had relatively small sample sizes which accounted for the skewed gender proportions and two studies did not report the gender of the participants. However, this gender imbalance is unlikely to affect results.^56^

No studies rated poorly on the MINORS quality assessment tool for follow-up studies, with scores ranging from 11 to 16 of 18. In general, retrospective studies rated poorer, possibly because the tool was not created specifically for retrospective studies, so although the criteria were modified, they may not be appropriate. According to the JBI tool for case studies, both studies were rated highly but study 1^43^ was of a higher quality than study 7.^20^ Although no studies were of poor quality, as mentioned above, none of the study designs produced high-quality evidence.

The 11 studies included in this review are likely to constitute all the available evidence, due to the inclusive search strategy and rigorous screening processes. The search strategy was checked by an independent expert in systematic reviews and screening of full texts was performed by two reviewers with 82% agreement. Chain searching revealed three new papers, but none were eligible for inclusion. The review was further strengthened by a second reviewer cross-checking the data extraction tables. Thus, conclusions arising from this review were based on all the available evidence.

Limitations of the review include the specificity of the eligibility criteria in relation to the mode of Bevacizumab administration. Ten papers described intravenous Bevacizumab and one study^6^ investigated intraarterial administration, making this paper noncomparable. Toxicities and tolerability were not evaluated as an outcome but as this information may be useful to clinicians, these were described for each study, but did not include information about specific side effects, which may also have been useful. Only 1 reviewer performed titles and abstracts screening. However, this was conducted very conservatively to ensure that all potential papers were included. We used validated quality assessment tools recommended by Ma et al.^40^ but the MINORS tool was not completely suitable for the retrospective studies, even after modification. This may have resulted in retrospective studies being scored lower quality than necessary. Additionally, quality assessment was conducted by 1 reviewer, which may have reduced the reliability of the results. However, no changes were made after cross-checking the results a week later. Finally, the exclusion of non-English language papers and not searching grey literature may have increased the possibility of selection bias. Although, this is unlikely as other language papers were not retrieved in the search, and gray literature was not searched due to the potential for issues with reliability and quality.^57^

## Conclusion and Recommendations

DIPG carries an extremely poor prognosis; children with DIPG have no chance of survival and likely a poor QOL. Bevacizumab has low toxicity and high tolerability, and the findings suggest a possible improvement in QOL and a reduction in steroid use in a selected group of patients. Although only 2 studies evaluated the role of Bevacizumab in treating radiation necrosis, results were promising in both. Due to insufficient evidence findings cannot be generalized, therefore, meaningful conclusions about Bevacizumab’s efficacy and suggestions for specific treatment cannot be recommended. The review highlights the need for further research specifically in relation to controlled prospective clinical trials with larger sample sizes, likely European or global studies, and which investigate various outcomes, in particular, QOL, clinical response, and steroid use. However, Bevacizumab is not a cure for DIPG, more effective therapies are desperately needed for this devastating disease.

## Supplementary Material

vdac100_suppl_Supplementary_Material
