# Supplementary material for: Does a Bevacizumab-based regime have a role in the treatment of children with diffuse intrinsic pontine glioma? A systematic review
Source: Neurooncol Adv. 2022 Jun 24;4(1):vdac100. doi: 10.1093/noajnl/vdac100 (PMC9270727; doi:10.1093/noajnl/vdac100)
Supplement: vdac100_suppl_Supplementary_Material [file vdac100_suppl_supplementary_material.doc]

Supplementary material

Supplementary Table S1.

| 23 | antiangiogenic activit*.mp. | 1607 |
| --- | --- | --- |
| 24 | anti-angiogenic activit*.mp. | 1343 |
| 25 | anti-angiogenic therap*.mp. | 1795 |
| 26 | 1 or 2 or 3 or 4 or 5 or 6 or 7 or 8 or 9 or 10 or 11 or 12 or 13 | 3036 |
| 27 | 14 or 15 or 16 or 17 or 18 or 19 or 20 or 21 or 22 or 23 or 24 or 25 | 47713 |
| 28 | 26 and 27 | 52 |

Results of an Ovid MEDLINE (1946 to September Week 2 2021) search which was carried out on 17/09/21:

| 1 | Diffuse Intrinsic Pontine Glioma/ | 101 |
| --- | --- | --- |
| 2 | diffuse intrinsic pontine glioma*.mp. | 547 |
| 3 | Brain Stem Neoplasms/ | 1837 |
| 4 | DIPG.mp. | 426 |
| 5 | pontine glioma*.mp. | 745 |
| 6 | diffuse midline glioma*.mp. | 145 |
| 7 | midline glioma*.mp. | 173 |
| 8 | brainstem glioma*.mp. | 490 |
| 9 | brain stem glioma*.mp. | 288 |
| 10 | brainstem tum*.mp. | 373 |
| 11 | brain stem tum*.mp. | 291 |
| 12 | brainstem neoplasm*.mp. | 17 |
| 13 | brain stem neoplasm*.mp. | 1848 |
| 14 | Bevacizumab/ | 12562 |
| 15 | bevacizumab.mp. | 16493 |
| 16 | avastin.mp. | 1364 |
| 17 | Angiogenesis Inhibitors/ | 26550 |
| 18 | angiogenesis inhibitor*.mp. | 28112 |
| 19 | antiangiogenic therap*.mp. | 2444 |
| 20 | antiangiogenic.mp. | 11646 |
| 21 | anti-angiogenic.mp. | 9635 |
| 22 | angiogenesis inhibitor*.mp. | 28112 |

Supplementary Figure S1.

Modified MINORS^34^ criteria:

1. A clearly stated aim: the question addressed should be precise and relevant in the light of available literature
2. Inclusion of consecutive patients: all patients potentially fit for inclusion (satisfying the criteria for inclusion) have been included in the study during the study period **and inclusion & exclusion criteria stated**
3. Prospective collection of data: data were collected according to a protocol established before the beginning of the study **and was followed (all outcomes to be measured reported)**
4. **Intervention standardised: all patients received the same treatment unless reasons stated otherwise**
5. Outcomes appropriate to the aim of the study: unambiguous explanation of the criteria used to evaluate the main outcomes which should be in accordance with the question addressed by the study. Also, the outcomes should be assessed on an intention-to-treat basis.
6. Unbiased assessment of the study outcomes: blind evaluation of objective outcomes and double-blind evaluation of subjective outcomes. Otherwise, the reasons for not blinding should be stated
7. Follow-up period appropriate to the aim of the study: the follow-up should be sufficiently long to allow the assessment of the main endpoint and possible adverse events
8. **No** loss to follow up **~~less than 5%~~**: all patients should be included in the follow up. Otherwise, the proportion lost to follow up should not exceed the proportion experiencing the major endpoint
9. Prospective calculation of the study size: information of the size of detectable difference of interest with a calculation of 95% confidence interval, according to the expected incidence of the outcome event, and information about the level for statistical significance and estimates of power when comparing the outcomes

The items are scored 0 (not reported), 1 (reported but inadequate) or 2 (reported and

adequate).

**Words in bold present changes made to original MINORS tool**

Supplementary Figure S2.

JBI critical appraisal checklist for case reports^36^:

1. Were patient’s demographic characteristics clearly described?
2. Was the patient’s history clearly described and presented as a timeline?
3. Was the current clinical condition of the patient on presentation clearly described?
4. Were diagnostic tests or assessment methods and the results clearly described?
5. Was the intervention(s) or treatment procedure(s) clearly described?
6. Was the post-intervention clinical condition clearly described?
7. Were adverse events (harms) or unanticipated events identified and described?
8. Does the case report provide takeaway lessons?

Questions are answered yes, no and not clear.

Supplementary Table S2.

Reasons for exclusion of full-text papers:

| **Author (year)** | **Labelled reason** | **Reason for exclusion** |
| --- | --- | --- |
| Couec (2012) | Wrong outcome | Doesn’t report any of the outcomes in inclusion criteria |
| Dahl (2019) | Wrong population | Analysis of outcomes wasn’t separate for patients with DIPG |
| Gupta (2018) | Wrong outcome  Wrong intervention | Bevacizumab not a primary treatment (surgical biopsy was) and doesn’t report any of the outcomes |
| Hoffman (2018) | Wrong intervention | Bevacizumab not a primary treatment, paper reports all treatments in long term survivors |
| Lee (2018) | Wrong population | Doesn’t include any patients with DIPG and all adults |
| Li (2015) | Wrong population | Unclear whether brainstem lesion was DIPG |
| Moriya (2018) | Wrong population | Didn’t specify whether brainstem tumour was DIPG |
| Narayana (2010) | Wrong population | Patients diagnosed with high grade glioma |
| Narayana (2012) | Wrong population | Patients diagnosed with high grade glioma, DIPG not mentioned |
| Parekh (2011) | Wrong population | All patients had high grade gliomas |
| Piha-Paul (2014) | Wrong outcome | Doesn’t report any of the outcomes in inclusion criteria and analysis of PFS wasn’t separate in patients with DIPG |
| Reismüller (2010) | Wrong outcome | Doesn’t report any outcomes in inclusion criteria |
| Reithmeier (2012) | Wrong population | Paper reports one adult patient with an astrocytoma |
| Salloum (2015) | Wrong outcome | Doesn’t report any of the outcomes in inclusion criteria apart from time to progression but reported as a characteristic rather than an outcome |
| Wolff (2011) | Wrong intervention | Bevacizumab not a primary treatment, paper reports all treatments given as induction treatment |
| Wolff (2012) | Wrong intervention | Bevacizumab not a primary treatment, paper reports all treatments |
| Yonezawa (2021) | Wrong population | All adults and diagnosed with high grade gliomas |
